# Solar pacing of storm surges, coastal flooding and agricultural losses in the Central Mediterranean

**DOI:** 10.1038/srep25197

**Published:** 2016-04-29

**Authors:** David Kaniewski, Nick Marriner, Christophe Morhange, Sanja Faivre, Thierry Otto, Elise Van Campo

**Affiliations:** 1 Université Paul Sabatier-Toulouse 3, EcoLab (Laboratoire d’Ecologie Fonctionnelle et Environnement), Bâtiment 4R1, 118 Route de Narbonne, 31062 Toulouse cedex 9, France; 2 CNRS, EcoLab (Laboratoire d’Ecologie Fonctionnelle et Environnement), 31062 Toulouse cedex 9, France; 3 Institut Universitaire de France, Secteur Biologie-Médecine-Santé, 103 boulevard Saint Michel, 75005 Paris, France; 4 CNRS, Laboratoire Chrono-Environnement UMR6249, Université de Franche-Comté, UFR ST, 16 Route de Gray, 25030 Besançon, France; 5 Aix-Marseille Université, CNRS, UM 34, Europôle de l’Arbois BP80, 13545 Aix-en-Provence, France; 6 University of Zagreb, Faculty of Science, Department of Geography, Marulićev trg 19/II, 10 000 Zagreb, Croatia

## Abstract

Storm surges, leading to catastrophic coastal flooding, are amongst the most feared natural hazards due to the high population densities and economic importance of littoral areas. Using the Central Mediterranean Sea as a model system, we provide strong evidence for enhanced periods of storminess leading to coastal flooding during the last 4500 years. We show that long-term correlations can be drawn between storminess and solar activity, acting on cycles of around 2200-yr and 230-yr. We also find that phases of increased storms and coastal flooding have impacted upon mid- to late Holocene agricultural activity on the Adriatic coast. Based on the general trend observed during the second half of the 20^th^ century, climate models are predicting a weakening of Mediterranean storminess. By contrast, our new data suggest that a decrease in solar activity will increase and intensify the risk of frequent flooding in coastal areas.

There is a growing interest in predicting extreme weather and climate events^1^,^2^, because shifts in the frequency and magnitude of heat waves, heavy rainfall, drought, windstorms and storm surges impact upon the natural environment, and cultural and socio-economic systems, more than changes in global mean climate^3^. Coasts are key geographic areas because they lie at the interface of climate change. Within this context, much attention has been paid to seaboards that are directly threatened by global sea-level rise^4^ and recurrent flooding events^5^,^6^. While these zones represent only 10% of the earth’s total land area, humanity tends to concentrate along or near coasts. In the year 2000, about 63 million people lived in coastal flood-prone areas^7^ and global flood losses were estimated to be approximately US$6 billion per year (data for the year 2005)^5^. The population at risk from storm surge events and coastal flooding could reach 286 million in 2030, and, by 2060, affect up to 411 million people^7^, increasing global flood losses to US$52 billion with projected socio-economic change^5^. Enhanced flood exposure in coastal areas, and increased losses caused by catastrophes, is primarily driven by global sea-level rise during the past ∼100 years^4^, but also by an intensification of storm surges, engendering recurrent overwashing and inundation of low-lying areas^6^. The vulnerability of coasts and deltas^8^ has largely been accentuated by falling fluvial sediment supply to coastal areas^9^ and increased rates of land subsidence^10^, leading to saline intrusion and erosion in the face of climate extremes.

Focusing on the densely populated Mediterranean countries, more than a third of the total population lives in coastal areas and on deltas (up to 1000 people per km^2^), representing less than 12% of the surface area. This population at risk, that grew from 95 million in 1979 to 143 million in 2000, could reach 174 million by 2025^11^. In this sensitive region, climate change is expected to generate modifications in both precipitation patterns and the frequency of flooding^7^. Whether further changes in storm activity will occur is still debated and no consensus has been reached in this respect^12^. Concentrating on Croatia as a study model, storminess and flooding led to damages of around US$ 79 million per year for the period 1981–2010^13^,^14^. While storm activity is relatively well understood, few records of their long-term natural variability extend beyond the period of instrumental records or the last millennium.

Here, we investigate storm activity in the Mediterranean during the last 4500 years and its impacts on human economy, using palaeoecological changes resulting from coastal flooding of deltas. We selected the Central Mediterranean Sea (CMS) as this zone corresponds to one of southern Europe’s key tourism and recreational areas, and is a major maritime route for the transport of goods to central and southeastern Europe. Furthermore, the CMS is considered to be a hotspot of global climate change^15^,^16^. For instance, climate upheavals and storm surges represent a constant socio-economic and ecological threat to its coastal areas^12^, exemplified by the vulnerability of the eastern Adriatic coast^17^ and by the problems facing the city and the lagoon of Venice^18^.

## Storms in the Central Mediterranean Sea

In the CMS, the strongest south-easterly and northeastern winds, respectively the Sirocco and the Bora, strike the long semi-enclosed Adriatic basin from autumn to spring^12^,^19^. While the occurrence of these winds is a main trigger of coastal swell^19^, the larger and quite persistent rises in sea level are produced by the sirocco-wind, which accumulates water at the closed northern end of the basin^12^,^19^. Associated cyclones force variations in the sea surface (termed *storm surges*) and follow basin-wide oscillations (termed *seiches*)^20^. Extremely high sea levels/high-surge events may happen, causing flooding of the North Adriatic coast (termed *acqua alta*, literally “high water”). These high-water events are primarily caused by enhanced south-easterly winds blowing over the shallow sea concurrently with a pronounced air-pressure gradient across the CMS. While, over most of the Adriatic basin, this south-easterly wind always prevails, there is a tendency for a strong Bora (“Dark Bora”) to occur before the peak of the surge, being replaced by the Sirocco during the most intense phase of the event^12^. Bora winds^21^,^22^ regularly generate gyres in surface coastal waters, depending on where the Bora’s strongest offshore jets occur^23^,^24^. The sea-surface gyres, resulting from wind stresses, push waters westward and eastward in the Adriatic basin^25^. The following component, which controls whether the western or the eastern coast is severely flooded, is dependent on the longitudinal wind, the Sirocco^17^. A relationship is therefore suggested between the storm waves and the flood events. The tide level and the principal Adriatic *seiche* also contribute considerably to the high-water events^20^,^26^.

## Results

### Study area

**Figure 1:**
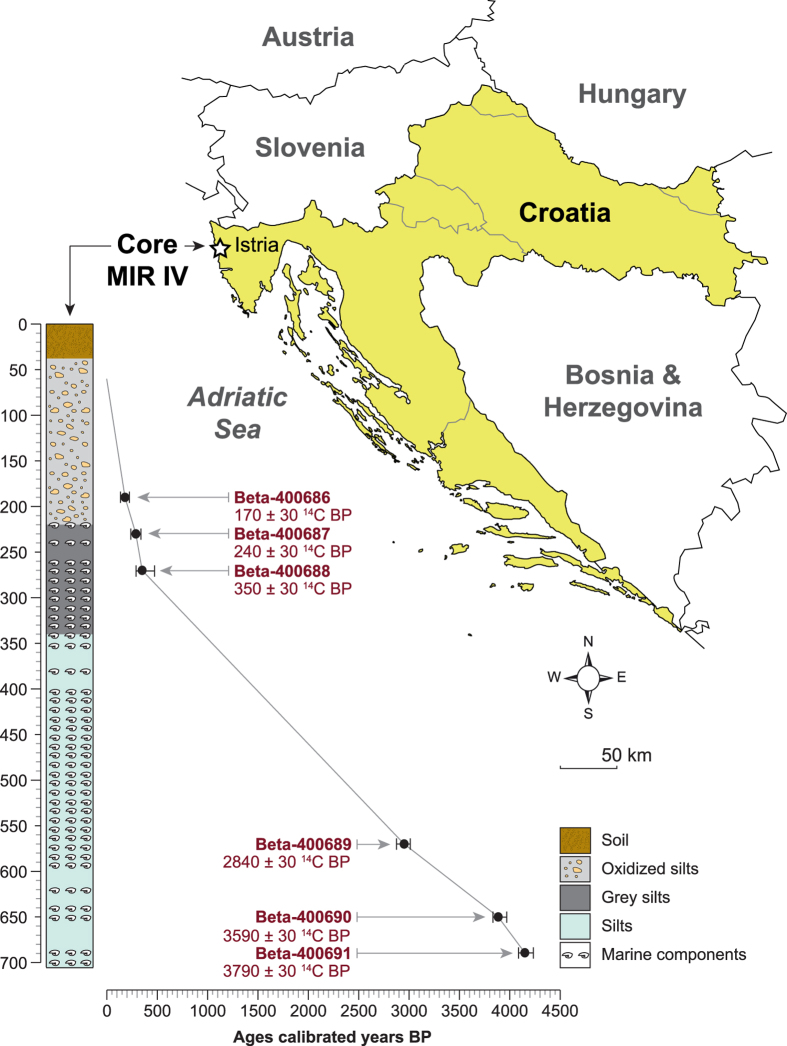
Geographical location of the study area in Croatia and radiocarbon chronology. The study site is denoted by a white star on the Istrian peninsula, coastal Croatia. The lithology of the core, with the influence of marine components, is detailed according to depth. The radiocarbon dates are depicted as intercepts and 2-sigma calibrations (95% of probability). The map is an original document drawn using Adobe Illustrator CS5 (http://www.adobe.com/fr/products/illustrator.html).

Terrestrial and marine biological indicators, used as proxies for storminess during the last 4500 years, were extracted from a 720-cm continuous core (MIR IV, 45°20′11.55″N, 13°39′30.29″E; +1 m MSL) drilled on the delta of the Mirna River (Gulf of Venice) in coastal Croatia (Fig. 1). The chronology of the core is based on accelerator mass spectrometry ^14^C dates of short-lived terrestrial samples (seeds and small leaves; Supplementary Table S1). No botanical macro-remains were found in the middle core but the use of marine shells (involving the radiocarbon-dating reservoir effect) and bulk samples (involving potential contaminants) were strictly avoided in order to minimize chronological biases in the age-depth model. Dated samples were calibrated [1-sigma (σ) and 2σ calibrations, respectively 68% and 95% of probability] using CALIB REV 7.0.4^27^. The average chronological resolution for the core stratigraphy is 7 years per cm^−1^ (1.43 mm per yr^−1^).

### An ecologically-based storm proxy for the CMS

**Figure 2:**
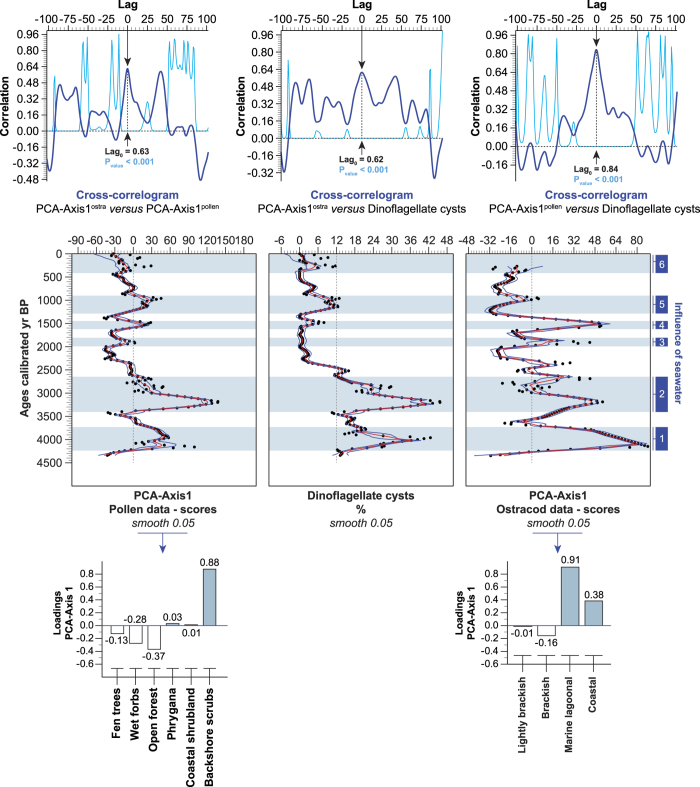
Proxy-based storm series for the last 4500 years. The three independent proxies (pollen, ostracods and dinoflagellate cysts) are depicted as two PCA-Axes1 (with the loadings), and as percentages. The PCA-Axis1^pollen^, PCA-Axis1^ostra^ and dinoflagellate cyst time-series are shown as LOESS smoothing plotted on a linear age-scale (ages are in calibrated years BP). The influence of seawater is underscored by the blue lines and numbered from 1 to 6. At the top, the cross-correlograms show the correlations between the PCA-Axis1^pollen^, the PCA-Axis1^ostra^, and the dinoflagellate cysts. Vertical axes show correlation coefficients while horizontal axes show the lag (1 unit = 1 sample). Significance level *P* = *0.05*.

Storminess was reconstructed using three independent biological proxies: pollen-based terrestrial ecosystems, ostracods and dinoflagellate cysts (Fig. 2). While inland penetration of seawater is attested by the intrusion of marine components, terrestrial ecosystems are also good markers of coastal flooding in the CMS. Storm-based coastal flooding generates the intrusion of saline water into the freshwater-fed plains, raising salinity in the hinterland, leading to land fragmentation by salt encroachments. These are attested by the impacts on coastal ecosystems. Amplified ecological erosion of coastal wetlands suggests repetitive intrusions of seawater, with the salinity and duration of flooding acting as the main pressures. This process led to a lowered protection of the shorelines previously ensured by these wetlands^28^,^29^. Salt-water intrusion also affects the groundwater table and can impact inland ecosystems^4^,^30^.

The cluster analysis (CA) has defined a *backshore scrubs* pollen-derived vegetation pattern (^Pd^VP; Supplementary Figs S1 and S2), which fits with the modern shoreline vegetation^31^,^32^, and which corresponds to the main loading (+0.88) in the principal component analysis (PCA) based on ^Pd^VPs (PCA-Axis1^pollen^; Fig. 2). The PCA-Axis1^pollen^ time series (77.64% of the total inertia) is positively correlated (Lag_0_ + 0.63, P_value_ < 0.001) with the PCA-Axis1^ostra^ scores (68.41% of the total inertia; based on ostracods; Supplementary Fig. S2) which are loaded by *marine lagoonal* (+0.91) and *coastal* (+0.38) ostracod assemblages in positive deviations (Fig. 2). The PCA-Axis1^pollen^ and PCA-Axis1^ostra^ are also correlated with increases in marine dinoflagellate cysts (Lag_0_ + 0.84 and +0.62, P_value_ < 0.001). The three signals (PCA-Axis1^pollen^, PCA-Axis1^ostra^, dinoflagellate cysts) are correlated and defined as periods when sea floods penetrated inland (Fig. 2).

**Figure 3:**
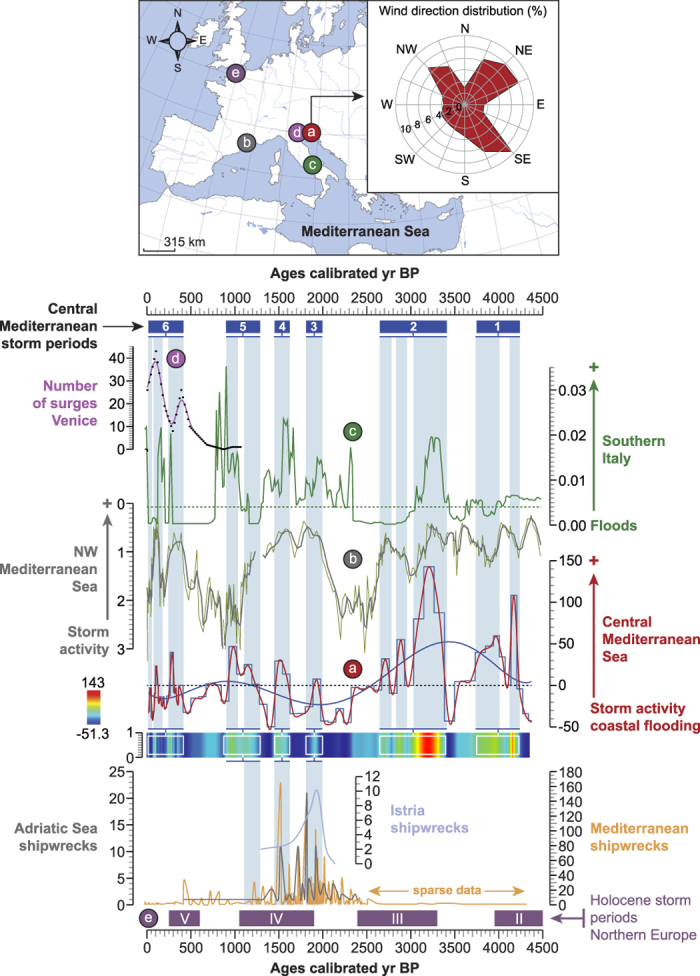
Reconstructed storm activity and coastal flooding in the Central Mediterranean for the last 4500 years (**a**). Storm activity, shown as PCA-Axis1 with a polynomial fitting curve and matrix plot, is compared to storminess in the northwest Mediterranean^35^ (**b**), extreme hydrological events in southern Italy^34^ (**c**), sea surges in Venice^39^, and storminess in Northern Europe^36^ (**e**). The blue-shaded lines highlight the most severe events recorded in the Central Mediterranean Sea, termed “storm periods” (numbered from 1 to 6). The map is an original document drawn using Adobe Illustrator CS5 (http://www.adobe.com/fr/products/illustrator.html).

Storm activity (detected by coastal flooding) in the Adriatic Sea was reconstructed using three defined patterns (*Coastal environment*, *Inputs of freshwater* and *Storm cluster*; Supplementary Fig. S2) in order to run a multi-proxy PCA. The resulting PCA-Axis 1 was plotted on a linear age-scale (Fig. 3). The PCA-Axis 1 loadings (77.06% of the total inertia) attributed negative values to the clusters *Coastal environment* (−0.015) and *Inputs of freshwater* (−0.208), whereas positive scores are only loaded by the *Storm cluster* (0.978). PCA-Axes 2 and 3 account for 16.34% and 6.60% of total inertia, respectively.

### Reconstructed storm activity

Enhanced storminess and coastal flooding in our CMS record, shown as positive deviations of the PCA-Axis 1, has been recorded during six intervals at 4250-3750, 3400-2550, 2000-1800, 1650-1450, 1300-900 and 400-100 calibrated years Before Present (cal yr BP), with peaks and intermediate periods of low activity (Fig. 3). The amplitude of the storm phases appears to be on the decrease since 3000 cal yr BP, supporting the TIMESLICE simulations that suggest that the Mediterranean surface storm track of the early-to-middle Holocene (12,000 to 6,000 years BP) may have been stronger than that recorded under preindustrial conditions^33^. The decreased intensity of the recorded storm events in the Adriatic may also result from the progradation of the river deltas during the last 3000 years^9^. These changes in coastal configuration could have increasingly sheltered the core site from storm activity.

When compared with the extreme hydrological events diagnosed in fluvial stratigraphy in southern Italy^34^ and storm activity in the northwest Mediterranean^35^ for the same period, the PCA-Axis 1 curve reveals in-phase events (Fig. 3), suggesting similar forcing factors operating at a wider Mediterranean scale. Our data also fit with periods of enhanced storminess in northern Europe^36^ (Fig. 3) and partly with the storm activity reconstructed in Bagnas lagoon, southern France^37^. For written records of sea surges at Venice^38^,^39^, we smoothed the original data using a 100-yr window and found that the two main peaks at 350 and 100 cal yr BP fit with enhanced storm activity from our record (Fig. 3). Furthermore, shipwreck data from the Adriatic and wider Mediterranean region^40^,^41^ present significant peaks around 2000-1800, 1650-1450 and 1300-900 cal yr BP that can be correlated with heightened periods of storminess in the CMS (Fig. 3), suggesting an impact of increased storminess on maritime trade.

### Periodicities of storminess

**Figure 4:**
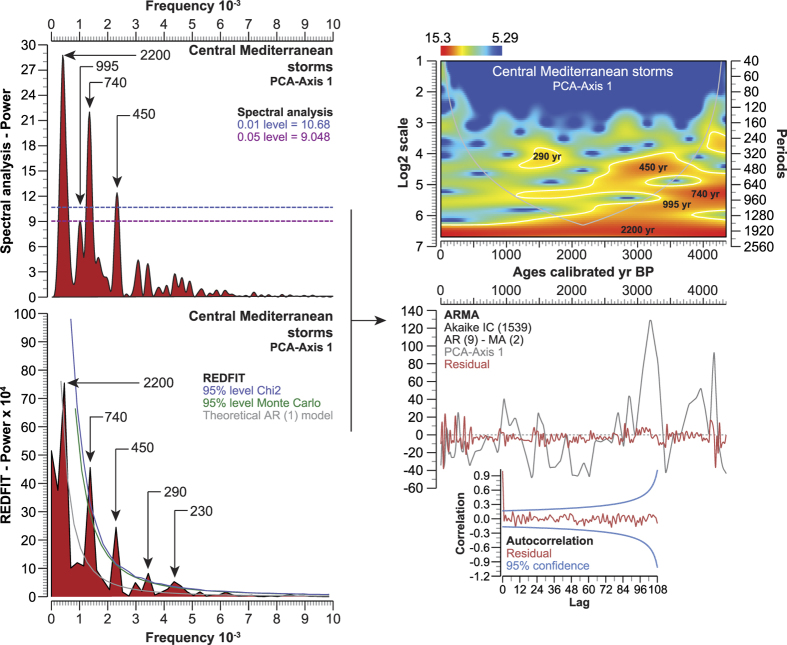
Periodicity of storminess in the Central Mediterranean during the last 4500 years. The periodicity of storminess is shown as a spectral analysis (Lomb periodogram) and REDFIT spectral analysis. The 0.01 and 0.05 significance levels (white noise, Lomb periodogram) are depicted as blue and purple lines. The 0.05 significance levels (Chi2 and Monte Carlo) for the REDFIT spectral analysis are depicted on the graph (blue and green lines). The time series is fitted to an AR (1) red noise model (grey line). The wavelet transform (scalogram) for the Central Mediterranean storminess is detailed. The cone of influence is depicted as a grey line, and the significance level (*P* = *0.05*) as white lines. The residual was extracted using an ARMA analysis and its periodicity tested by autocorrelation (95% confidence).

Three main periodicities elucidated in the storm signal are centered on 450-yr, 740-yr and ~2200-yr. These cycles are confirmed by the Lomb periodogram and the scalogram, corrected by the REDFIT spectral analysis (Fig. 4). Three extra-bands, with lower powers, were also recorded (995-yr, 290-yr, 230-yr). The PCA-Axis1 residual has no influence on the defined periodicities as shown by the autocorrelation applied to the ARMA data (Fig. 4).

**Figure 5:**
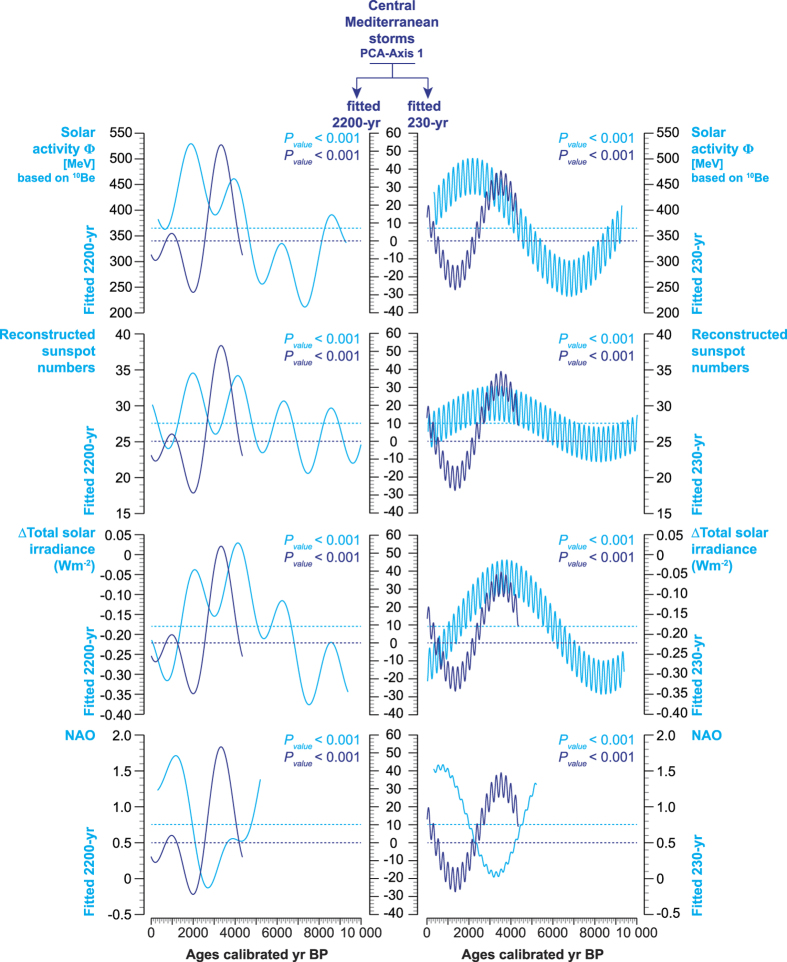
Long-term trends in storm surges, solar activity and the NAO. Sinusoidal regressions show the longer (fitted to 2200-yr) and shorter (fitted to 230-yr) periodicities defining the long-term trends in solar activity^42^,^43^,^44^, the NAO^48^ and storm surges.

The periodicity of storm activity and coastal flooding closely mirrors that of solar activity (Supplementary Fig. S3) reconstructed from total solar irradiance (∆TSI)^42^, sunspot numbers^43^ and from the solar modulation function Φ [MeV] based on cosmogenic radionuclides (^10^Be production rates)^44^. This suggests that periodic solar irradiances, which have played a key role in Holocene climate dynamics^45^, may also have been a trigger of severe storm events and coastal flooding in the CMS (Fig. 3). When the signals are fitted to 2200-yr and 230-yr filters, storm activity is mainly in-phase with solar minima (Fig. 5). A connection between low solar irradiance (mostly for the winter period) and the Northern Atlantic Oscillation (NAO) has been suggested for the period 1001–1860 Current Era (CE), but with a time lag of approximately 40 years (or more) for the response of the NAO^46^,^47^. This relationship is also indirectly supported by our storm data for the last 4500 years that also reveals an anti-phase periodicity with the NAO^48^ (Fig. 5 and Supplementary Fig. S4).

### Agricultural losses

**Figure 6:**
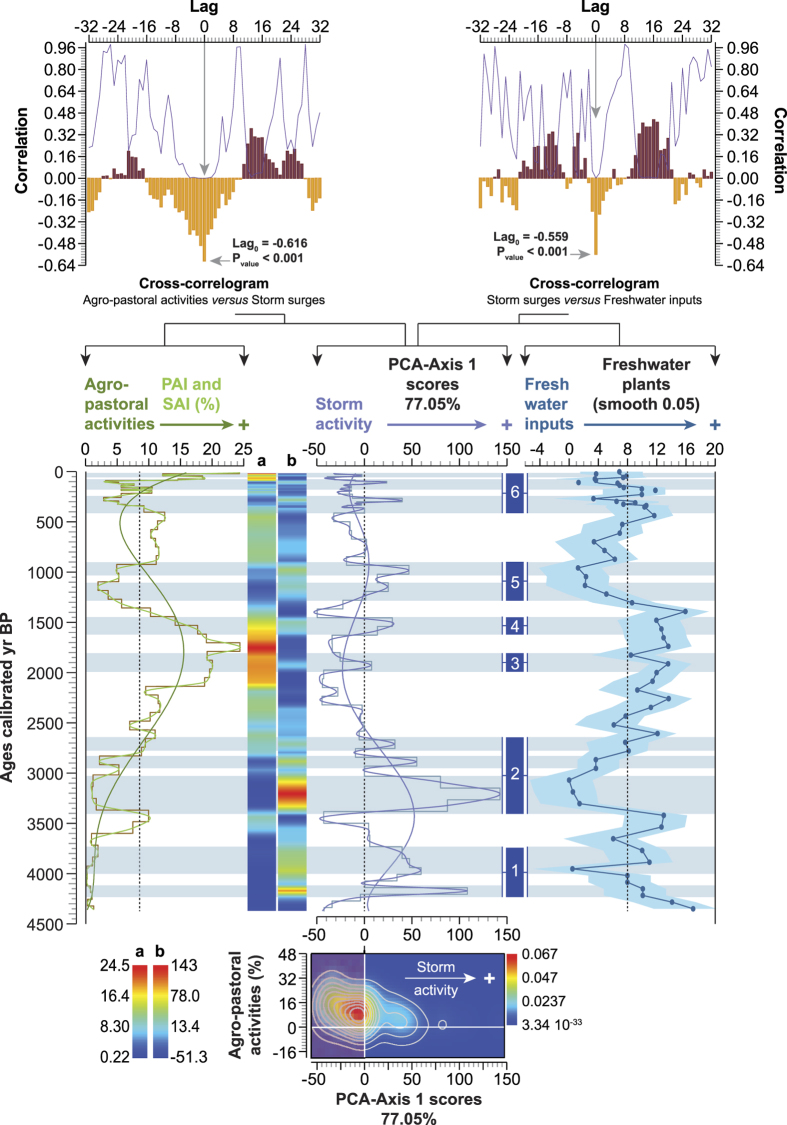
Storm activity, coastal flooding, freshwater inputs and agricultural productivity for the last 4500 years. Agro-pastoral activities (PAI and SAI) are plotted on a linear age-scale (with a polynomial fitting curve and a matrix plot) and compared with storminess. The freshwater inputs are depicted using a LOESS smoothing curve plotted on a linear age-scale. The blue lines highlight the most severe storms recorded in the Central Mediterranean Sea. At the top, the cross-correlograms show the link between storm surges, agro-pastoral activities and freshwater inputs with the correlation coefficient at Lag_0_ and the associated P_value_. Vertical axes show correlation coefficients while horizontal axes show the lag (1 unit = 1 sample). Significance level *P* = *0.05*. At the bottom, the relationship between the storm surges and the agro-pastoral activities was analysed using a Kernel density map (*Radius* 1.078).

**Figure 7:**
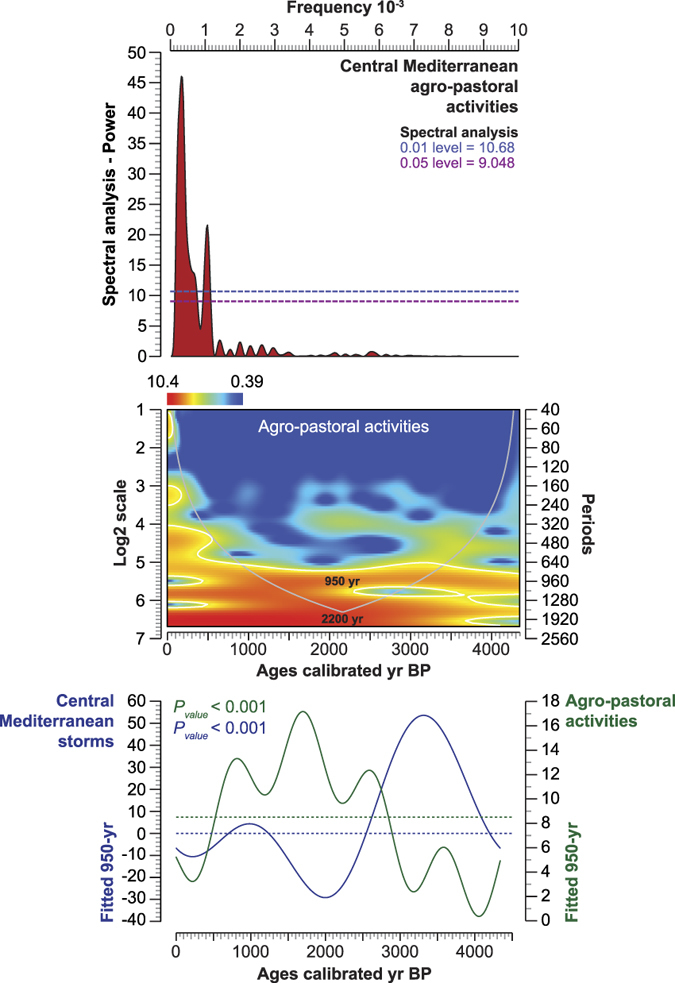
Periodicity of agro-pastoral activities in the Central Mediterranean for the last 4500 years. The periodicity of agro-pastoral activities is shown as a spectral analysis (Lomb periodogram). The 0.01 and 0.05 significance levels (white noise, Lomb periodogram) are depicted as blue and purple lines. The wavelet transform (scalogram) for agro-pastoral activities is detailed. The cone of influence is depicted as a grey line, and the significance level (*P* = *0.05*) as white lines. The sinusoidal regression shows the long-term trends in agro-pastoral activities *versus* storm activity (fitted to 950-yr).

To evaluate the agricultural losses that may arise from coastal flooding by storm events, an *agro-pastoral activities*^Pd^VP was elaborated using anthropogenic indicators (Supplementary Fig. S5). We compared and contrasted this record with the reconstructed storm activity (Fig. 6). The PCA-Axis1 is negatively correlated with the *agro-pastoral activities*^Pd^VP (Lag_0_ − 0.616, P_value_ < 0.001) and is also negatively correlated with the *freshwater plants*^Pd^VP (Lag_0_ − 0.559, P_value_ < 0.001). This suggests that low storm activity and enhanced freshwater inputs in the delta have favoured arboriculture and agriculture. Inversely, periods of higher storm surges, which generated the intrusion of saline water into the freshwater-fed plains and into the groundwater table, led to severe agricultural losses. This assumption is strengthened by the Kernel density map, which shows that anthropogenic activities are mainly concentrated during periods of low storm activity (Fig. 6). The cyclicity of storminess and coastal flooding in the CMS has induced a periodicity in agro-pastoral activities with a main 950-yr cycle (Fig. 7). The comparison of the two signals, fitted to a 950-yr filter (Fig. 7), shows that, at a millennial time-scale, anthropogenic activities and storminess are in anti-phase (Lag_0_ − 0.564, P_value_ < 0.001).

## Discussion

During the last 4500 years, extremely high-surge events happened during six periods (Fig. 3), causing flooding of the North Adriatic coast. The three proxies (Supplementary Fig. S2) mark the occurrence of wind-driven waves capable of penetrating the coastal strip for long periods (from hours to months) but with different intensities. The coupled SWAN + ADCIRC model has already shown the complementary roles of wind-driven waves and circulation processes in the occurrence of storm surges in coastal areas^49^. During the severe storm periods termed 1 and 2 (Fig. 3), the amplitude of the three signals is similar (Fig. 2 and Supplementary Fig. S2), with significant agricultural losses inferred from our pollen data (Fig. 6). These events can only result from unusually high sea level (high-tide peaks or prolonged deviations from mean sea level), stemming from pronounced wind stresses that push and accumulate waters at the closed northern end of the basin. It has been previously shown that the occurrence of these winds, which mediate the wave climate^50^, are a main trigger of coastal swell^19^. By contrast, the interval 2500-100 cal yr BP appears quite different. During a first period (storms termed 3 and 4), the storm signal is weaker (increase in marine-lagoonal ostracods with a reduced development of backshore scrubs and a low input of dinoflagellate cysts; Fig. 2 and Supplementary Fig. S2), which suggests the occurrence of weakened winds leading to storms of lower severity in the CMS. This drop in storm intensity is confirmed by their low impacts on agricultural yields (Fig. 6). The storm periods 5 and 6 are marked by repetitive intrusions of seawater onto the delta, affecting both the freshwater plains and the groundwater table as shown by the enhanced erosion of coastal ecosystems (high growth of backshore scrubs) and the agricultural losses, but with a gradual rise in freshwater influence since 900 cal yr BP (Fig. 6).

Focusing on long-term trends, the storm activity and coastal flooding shows important similarities with solar periodicities (Supplementary Fig. S3) and seems to be reinforced during periods of lower solar radiation. It has been suggested, focusing on the 11-yr solar cycle for the 1948–2008 CE period, that solar activity affected the frequency of high‐surge events in Venice by modulating the spatial patterns of the key modes of atmospheric circulation, the favorable patterns for high‐surge events, and their mutual connections, so that interactions are heightened during solar maxima and inhibited during solar minima^51^. By contrast, Camuffo *et al.*^39^ have suggested, based on data for the last millennium, that while a maximum frequency of storm activity occurred in the Adriatic during the Spörer Minimum, sunspot series yield weak correlations for the other phases of low activity (e.g. Oort Minimum, Wolf Minimum, and Maunder Minimum). This suggests that a teleconnection between storms and sunspots is unlikely in this region. Furthermore, no teleconnection was detected between Venice surges and the NAO^39^.

Our data, focused on long-term trends of severe storms that led to flooding (Fig. 5), suggest enhanced storm activity during solar minimum and low (or negative) NAO scores for the last 4500 years. It has been previously shown that when ∆TSI decreased, more northern-latitude terrestrial regions faced cooler climate^52^. Low ∆TSI scores induce shifts in the stratospheric ozone that engender a cooling of the stratosphere and land-surface temperatures^52^. The atmospheric responses to low ∆TSI lead to synchronous intensifications in North Atlantic drift ice, decreases in North Atlantic deep-water intensity, cooling of the high-latitude continent and ocean surface, and activate the negative mode of the Arctic Oscillation/NAO^46^. Low and negative NAO may have affected storminess in the CMS during winter through an intensification of winds as previously shown for the first half of the 20^th^ century^53^. A similar connection between low NAO and storminess has also been suggested in the Azores region^54^. Based on long-term trends, the recorded flood events in the CMS are obviously linked to low solar activity through the modulation of the NAO, a reinforcement of winds, and storm waves (Fig. 3).

These assumptions are supported by the severe storm surges that led to disastrous floods in Italy and Croatia^55^, respectively on the 4^th^ November 1966 and 1^st^ December 2008^17^. These events occurred during intervals marked by low solar activity (cycle 19–20 minimum, and cycle 23–24 minimum), and negative scores of the NAO index (−0.68/−0.18 for early November 1966, and −0.32/−0.28 for early December 2008). The measured annual value for the NAO is −3.94 for 1966, and −4.54 for 2008^56^. A strong Bora-wind was recorded in both 1966 and 2008, with 36.0 m/s^−1^ as the maximum wind speed in each case. The maximum 10-minute wind speed at 35 m above ground level reached 30.1 m/s^−1^ in 2008^57^. The offshore wave heights reached 8 m (1966) and 3.2 m (2008) according to data recorded 15 km off the Venetian coast^55^. The main difference between 1966 and 2008 is the Sirocco velocity^17^, which was orientated towards the west (28 m/s^−1^ offshore) in November 1966 and towards the east (20.3 m/s^−1^ offshore) in 2008^55^. These two devastating episodes indicate that, while storm surges occurred several times per decade during the 20^th^ century CE in the CMS, the strongest events seem to be linked with low solar radiation and low/negative scores of the NAO. The mechanisms behind this process must be further investigated to establish the context for such occurrences and their aftermaths during solar minima.

## Conclusions

During the last five millennia, our data suggest that storm activity in the CMS occurred with various severities, differentially impacting coastal agriculture. For the near future, because urbanization rates and population growth in coastal areas already surpass the hinterland, mainly driven by rapid economic development^7^, a rise in the severity of winter storminess, induced by diminished solar activity^58^ after the grand solar maximum of the 20^th^ century CE^59^,^60^, could enhance coastal vulnerability by recurrent floods. This process will be accentuated by human-induced reductions in sediment supply to littoral areas, subsidence of clastic coasts and global sea-level rise. During the 21^st^ century, severe storm activity could be one of the dominant contributors to extreme flooding events and increased losses caused by natural disasters in the Mediterranean, contrasting with the scenarios proposed by climate models^61^.

## Methods

### Biological data

Samples were prepared for pollen analysis using the standard procedure for clay samples. Pollen frequencies (percentages) are based on the terrestrial pollen sum, excluding local hygrophytes and spores of non-vascular cryptogams. Aquatic taxa frequencies were calculated by adding the local hygrophytes-hydrophytes to the terrestrial pollen sum. The aquatic taxa scores, which attest to freshwater inputs onto the delta, were calculated by summing the local hygrophytes and hydrophytes. Dinoflagellate cysts (resting stage of marine plankton) were counted on pollen-slides and are displayed as concentrations (cysts per cm^3^ and percentages). Ostracods (Crustacea) were extracted from the same samples as the pollen and dinoflagellate cysts in order to avoid any analytical bias. The ostracofauna was picked from the sand fraction of the washed sediment (≤2 mm to ≥50 μm). On the basis of the species ecology outlined by Lachenal^62^, these were attributed to four groups: lightly brackish, brackish, marine-lagoonal and coastal (see Supplementary Raw Data). The dinoflagellate cysts and ostracods were used as independent proxies for marine flooding during storm events.

### Statistical analyses

All biological data were analyzed using the software package PAST, version 2.17c^63^. Pollen data were analysed using cluster analysis with *paired group* as the algorithm and *correlation* as the similarity measure (Supplementary Fig. S1). Cluster analysis was used to (i) compute the lengths of tree branches, using branches as ecological distances between groups of taxa (descending type), and (ii) categorize a salt-tolerant group. Each cluster was summed to create pollen-derived vegetation patterns (^Pd^VP), shown as boxplots (Supplementary Fig. S1), and plotted on a linear age-scale (Supplementary Fig. S2). The ostracod time-series is denoted as relative abundances, grouped according to their present-day ecology, and plotted on a linear age-scale (Supplementary Fig. S2). A second cluster analysis (*paired group* as algorithm and *correlation* as the similarity measure), performed on terrestrial and marine ecosystems (^Pd^VP, ostracods and dinoflagellate cysts), was implemented to generate a storm assemblage (Supplementary Fig. S2). The three defined patterns (“Coastal environment”, “Inputs of freshwater” and “Storm cluster”) were established by summing the corresponding cluster.

A first principal components analysis (PCA) was performed to test the ordination of terrestrial ecosystems by assessing major changes in the ^Pd^VP (Fig. 2). The “cultivated species-weeds” and “freshwater plants” assemblages were excluded from the matrix. The main variance is loaded by the PCA-Axis1 (termed PCA-Axis1^pollen^), which has been plotted on a linear age-scale. The same process was used for the ostracod time-series and a PCA-Axis1 (term PCA-Axis1^ostra^) was established (Fig. 2). The relationships between the PCA-Axis1^pollen^, the PCA-Axis1^ostra^, and the dinoflagellate cysts were analysed using linear detrended cross-correlations (_LD_CC, *P* = *0.05*). The _LD_CC assesses the time alignment of two time-series by means of the correlation coefficient. The series have been cross-correlated to ascertain the best temporal match and the potential lag between the two. The correlation coefficient is then plotted as a function of the alignment position (Fig. 2). Positive and negative correlation coefficients are considered, focusing on the Lag_0_ value (with +0.50 and −0.50 as significant thresholds). The PCA-Axis1^pollen^, PCA-Axis1^ostra^ and dinoflagellate cyst time-series have been plotted using a LOESS smoothing (with bootstrap and smooth 0.05) on a linear age-scale (Fig. 2).

The three defined patterns (“Coastal environment”, “Inputs of freshwater” and “Storm cluster”) were finally used to run a final PCA in order to reconstruct storm activity (by coastal flooding) in the Adriatic Sea. The resulting PCA-Axis 1 was plotted on a linear age-scale with a polynomial fitting curve and a matrix plot (individual scatterplot) (Fig. 3).

The periodicity of the storm signal (Fig. 4) was investigated using a wavelet analysis (wavelet transform) with Morlet as the basis function. A regular interpolation (20-yr) was initially run on the dataset. The scalograms are displayed as log2 scale and periods against a linear age-scale. The cone of influence marks the increasing importance of the edge effects. A spectral analysis was calculated to analyse the periodicity in terms of frequency/power (Fig. 4). A REDFIT analysis was performed to reduce the red noise and superimposed on the spectral analysis, with oversampling = 1, segments = 1, window = rectangular (Fig. 4). The potential effect of the residual on the periodicity was tested using ARMA analysis and autocorrelation (95% confidence). The main periodicities are highlighted by arrows on the graphs (Fig. 4). The same process was used for the solar time-series (solar activity Φ based on ^10^Be, ∆Total Solar Irradiance and sunspot numbers)^42^,^43^,^44^ and the NAO^48^. A regular interpolation (20-yr) was also run on each time-series before performing a REDFIT analysis and a wavelet transform (Supplementary Fig. S3).

Sinusoidal regressions were finally used in order to model periodicities in the time-series (the P_value_, based on an F test, gives the significance of the fit), in combination with spectral analysis. The larger (2200-yr) and the shorter (230-yr) periodicities were selected to highlight the long-term trends in solar activity^42^,^43^,^44^, NAO^48^ and storm surges (Fig. 5).

### Agro-pastoral activities

The agro-pastoral activities (Fig. 6), which correspond to primary (PAI) and secondary (SAI) anthropogenic indicators, are shown as dots and polynomial curves plotted on a linear age-scale (Supplementary Fig. S5). The cluster “cultivated species and weeds” was summed to create a ^Pd^VP (Fig. 6) that was also plotted on a linear age-scale with a polynomial fitting curve and a matrix plot (individual scatterplot). The relationship between the PCA-Axis1 and the ^Pd^VP was analysed using a _LD_CC (*P* = *0.05*) and a Kernel density map, with Gaussian as the Kernel function (*Radius* 1.078) (Fig. 6). The periodicity of agro-pastoral activities was investigated using a wavelet analysis (Fig. 7). A regular interpolation (20-yr) was initially run on the dataset. The scalograms are displayed as log2 scale and periods against a linear age-scale. A spectral analysis was calculated to analyse the periodicity in terms of frequency/power (Fig. 7). A sinusoidal regression was used, based on the 950-yr periodicity, to highlight the long-term trends in agro-pastoral activities *versus* storm surges (Fig. 7).

### Freshwater plants

The freshwater plants, consistent with freshwater inputs onto the delta, were summed to create a ^Pd^VP shown as LOESS smoothing (with bootstrap and smooth 0.05) plotted on a linear age-scale. The relationship between the PCA-Axis1 and the freshwater plants was analysed using _LD_CC (*P* = *0.05*; Fig. 6).

### Shipwreck database

Shipwreck data from the Mediterranean, the Adriatic and the Istrian coasts (Fig. 3) were compiled from two sources^40^,^41^.

## Additional Information

**How to cite this article**: Kaniewski, D. *et al.* Solar pacing of storm surges, coastal flooding and agricultural losses in the Central Mediterranean. *Sci. Rep.* **6**, 25197; doi: 10.1038/srep25197 (2016).

## Supplementary Material

Supplementary Information

Supplementary Dataset 1
