# Supplementary material for: Solar pacing of storm surges, coastal flooding and agricultural losses in the Central Mediterranean
Source: Sci Rep. 2016 Apr 29;6:25197. doi: 10.1038/srep25197 (PMC4850431; doi:10.1038/srep25197)

# Supplementary materials

## Solar pacing of storm surges, coastal flooding and agricultural losses in the Central Mediterranean

David Kaniewski[1,2,3Ψ], Nick Marriner[4], Christophe Morhange[3,5], Sanja Faivre[6], Thierry Otto[1,2], Elise Van Campo[1,2]

### Supplementary figures and table

**Supplementary Figure S1. Development of a backshore scrub assemblages.** Cluster analysis of the main taxa (%) from the core MIR IV calculated with paired group as the algorithm and correlation as the similarity measure. The pollen-types from each cluster (not including the freshwater plants and dinoflagellate cysts) were summed to create pollen-derived vegetation patterns. The pollen-derived vegetation patterns are shown as boxplots.

**Supplementary Figure S2. Development of a storm cluster in the Central Mediterranean for the last 4500 years**. The spindle diagram (cumulative %) depicts the dynamic of the three independent proxies in the coastal area. The cluster analysis was calculated with paired group as the algorithm and correlation as the similarity measure.

**Supplementary Figure S3. Periodicity of solar activity**. The periodicity of solar activity is shown as REDFIT spectral analysis. The 0.05 significance levels (Chi2 and Monte Carlo) are depicted on the graph (blue and green lines). The time series is fitted to an AR (1) red noise model (grey line). The wavelet transforms for solar activity[42-44] are denoted. The cone of influence is depicted as a grey line, and the significance level ($P = 0.05$) as white lines.

**Supplementary Figure S4. Periodicity of the NAO**. The periodicity of the NAO is using a REDFIT spectral analysis. The 0.05 significance levels (Chi2 and Monte Carlo) are depicted on the graph (blue and green lines). The time series is fitted to an AR (1) red noise model (grey line). The wavelet transforms for the NAO[48] are detailed. The cone of influence is denoted as a grey line, and the significance level ($P = 0.05$) as white lines.

**Supplementary Figure S5. Agro-pastoral activities in coastal areas for the last 4500 years**. The agro-pastoral activities, shown as percentages and polynomial fitting curves, are plotted on a linear age-scale. The chronology is expressed as calibrated year BP and calibrated year Current Era (CE) - Before Current Era (BCE).

**Supplementary Table S1.** Details of the $^{14}C$ age determinations for the core MIR IV.

**Supplementary Data.** Data used in this study.

**Supplementary Table S1.** Details of the $^{14}C$ age determinations for the core MIR IV.

| | | | **Calibrated dates BP** | | **Intercepts** |
|---|---|---|---|---|---|
| | **Depth (cm)** | **$^{14}C$ yr BP** | **1σ-68%** | **2σ-95%** | **BP** |
| Beta-400686 | 190 | 170±30 | 220-165 | 230-135 | 185 |
| Beta-400687 | 230 | 240±30 | 310-280 | 320-270 | 295 |
| Beta-400688 | 270 | 350±30 | 380-320 | 415-315 | 350 |
| Beta-400689 | 570 | 2840±30 | 2990-2920 | 3035-2865 | 2955 |
| Beta-400690 | 650 | 3590±30 | 3925-3850 | 3975-3835 | 3890 |
| Beta-400691 | 690 | 3790±30 | 4235-4195 | 4255-4085 | 4155 |

## Supplementary Figure S1

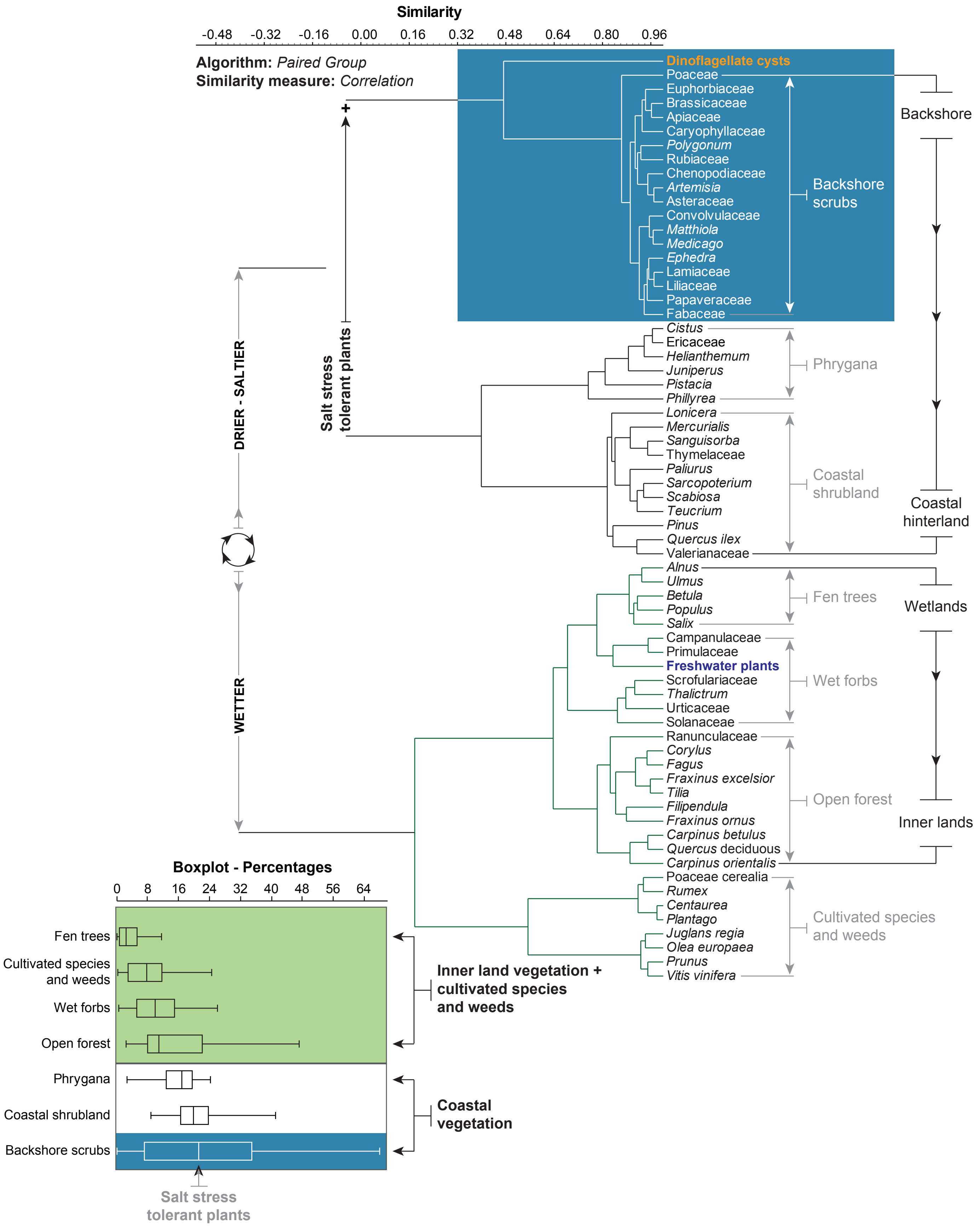

## Supplementary Figure S2

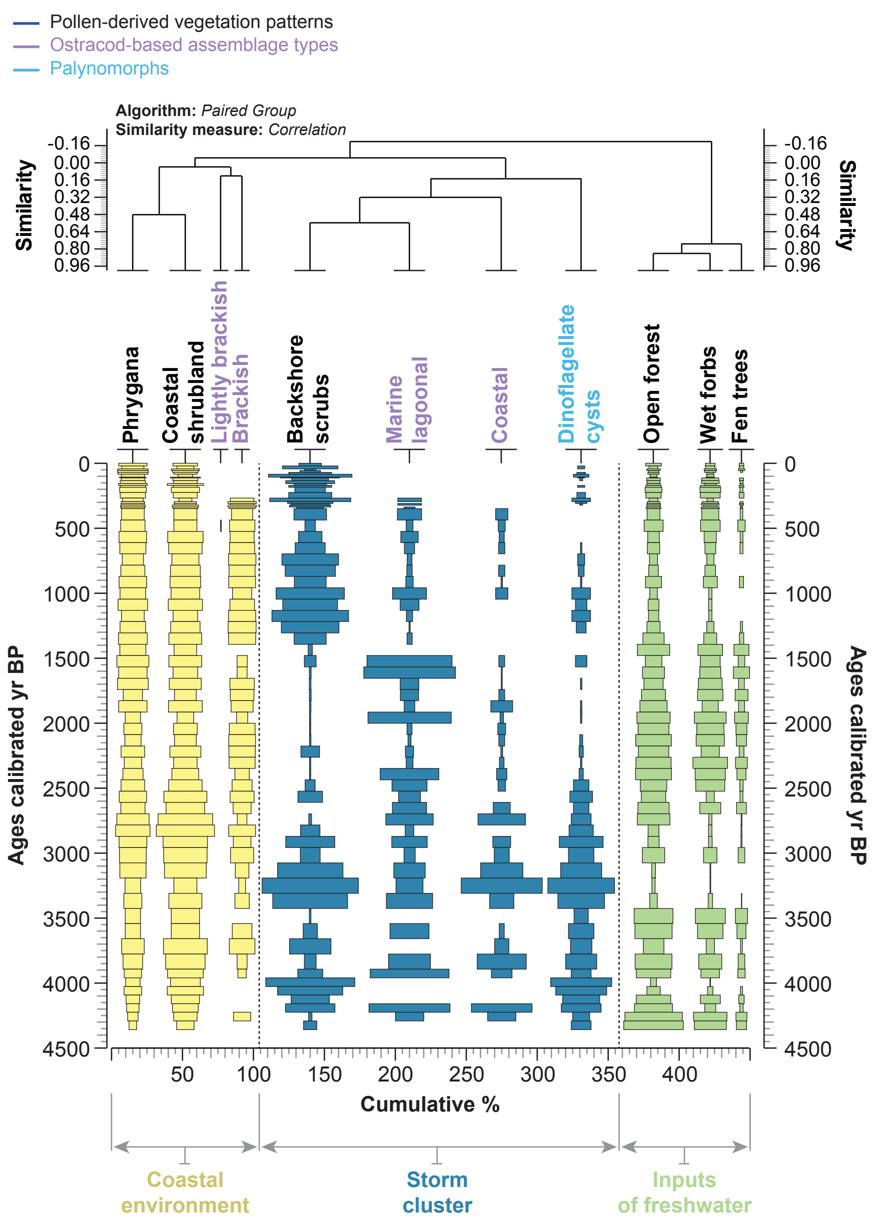

# Supplementary Figure S3

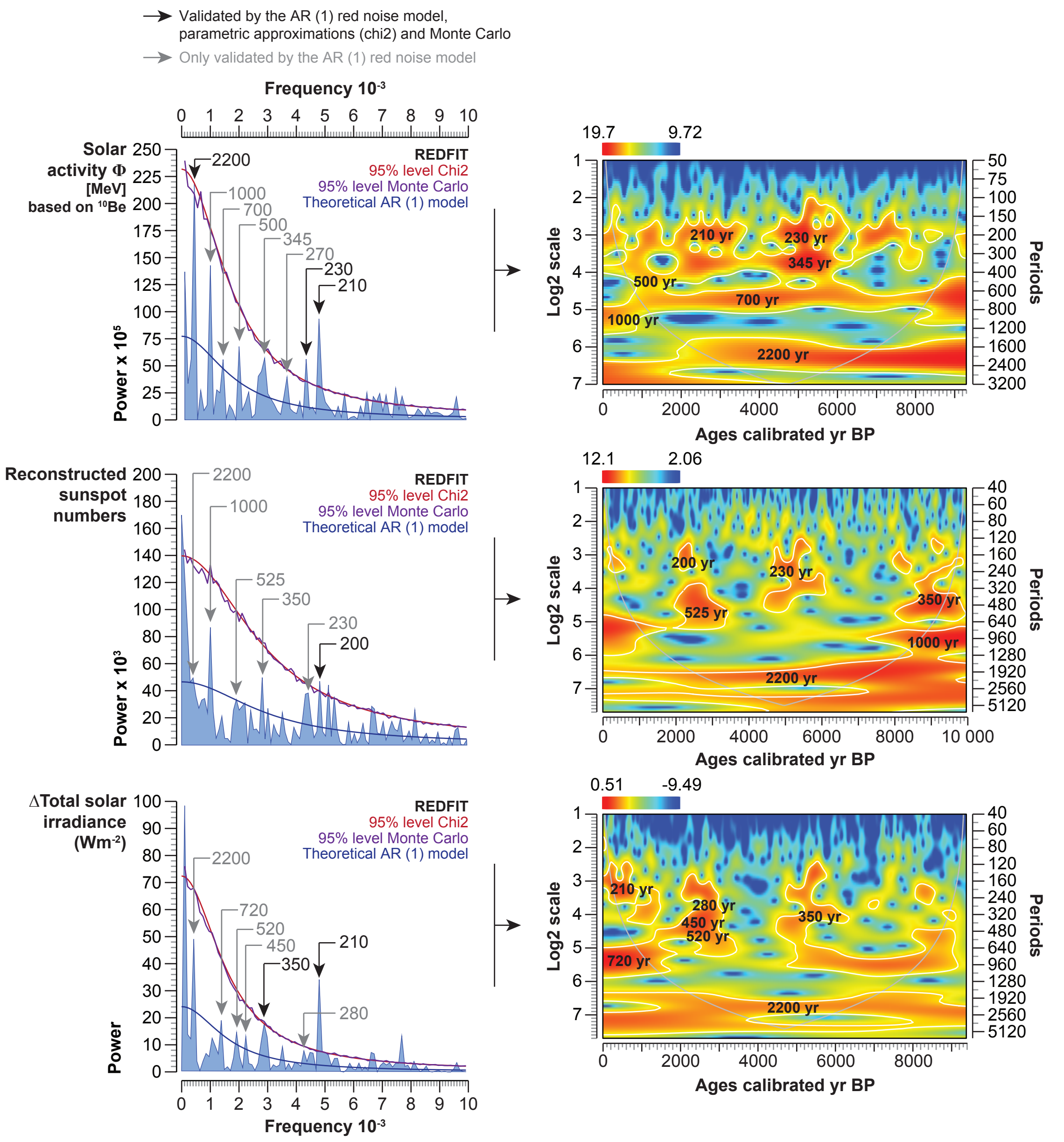

## Supplementary Figure S4

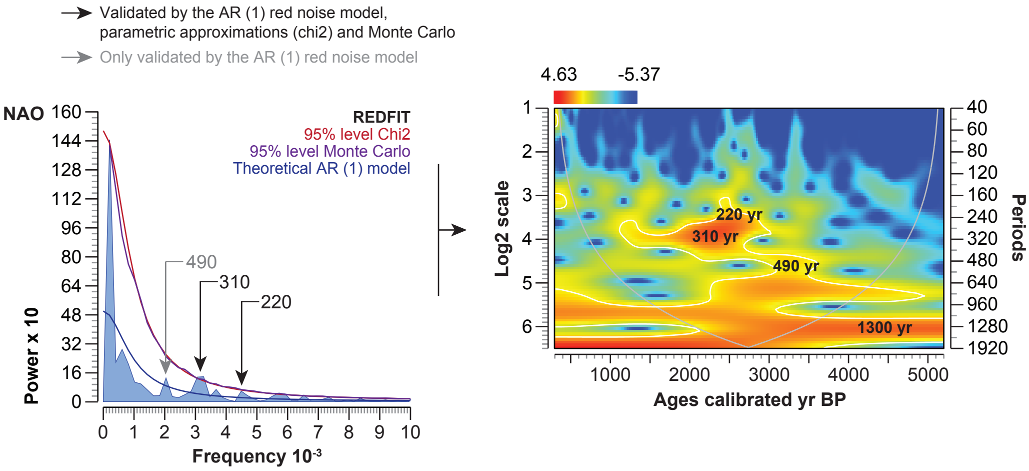

## Supplementary Figure S5

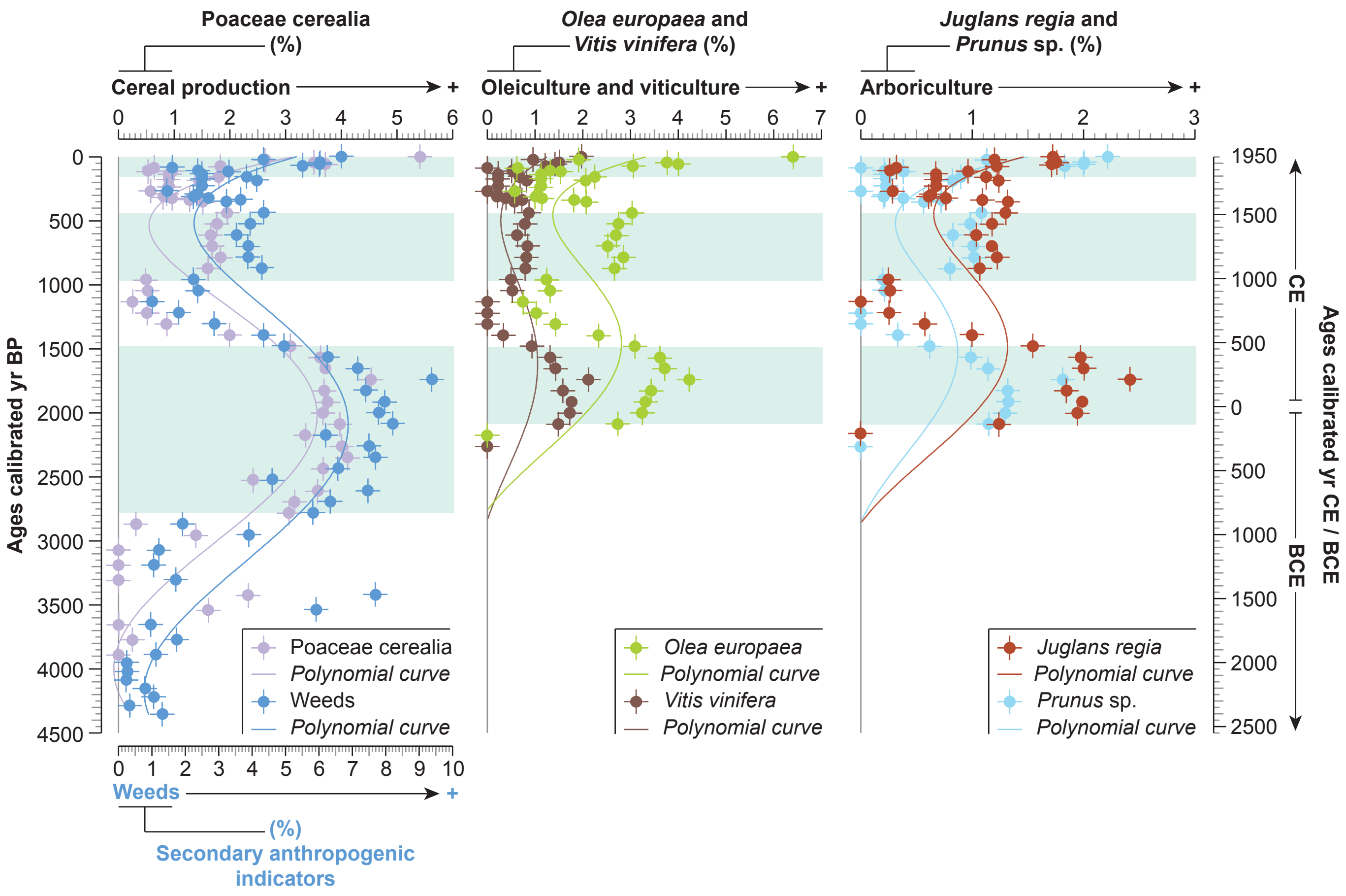

Supplement: Supplementary Information [file srep25197-s1.pdf]
